# The undebated issue of justice: silent discourses in Dutch flood risk management

**DOI:** 10.1007/s10113-016-1086-0

**Published:** 2016-12-16

**Authors:** Maria Kaufmann, Sally J. Priest, Pieter Leroy

**Affiliations:** 1 Institute for Management Research, Radboud University, Thomas van Aquinostraat, P.O. Box 9108, 6500 HK Nijmegen, The Netherlands; 2 Flood Hazard Research Centre, Middlesex University, London, UK

**Keywords:** Justice, Flood risk management, The Netherlands, Discursive institutionalism, Discourses

## Abstract

Flood risk of all types of flooding is projected to increase based on climate change projections and increases in damage potential. These challenges are likely to aggravate issues of justice in flood risk management (hereafter FRM). Based on a discursive institutionalist perspective, this paper explores justice in Dutch FRM: how do institutions allocate the responsibilities and costs for FRM for different types of flooding? What are the underlying conceptions of justice? What are the future challenges with regard to climate change? The research revealed that a dichotomy is visible in the Dutch approach to FRM: despite an abundance of rules, regulations and resources spent, flood risk or its management is only marginally discussed in terms of justice. Despite that, the current institutional arrangement has material outcomes that treat particular groups of citizens differently, depending on the type of flooding they are prone to, area they live in (unembanked/embanked) or category of user (e.g. household, industry, farmer). The paper argues that the debate on justice will (re)emerge, since the differences in distributional outcomes are likely to become increasingly uneven as a result of increasing flood risk. The Netherlands should be prepared for this debate by generating the relevant facts and figures. An inclusive debate on the distribution of burdens of FRM could contribute to more effective and legitimate FRM.

## Introduction

Notions of fairness, equity and justice are increasingly being discussed in the context of environmental issues such as climate change (e.g. Adger 2001; Ikeme 2003; Heyward 2007) and, in particular, in relation to flood risk management (FRM) (e.g. Johnson et al. 2007; Walker and Burningham 2011). Concerns about just, fair or equitable FRM are acknowledged by the EU Floods Directive (2007), which states ‘The solidarity principle is very important in the context of FRM… Member States should be encouraged to seek a fair sharing of responsibilities’ (Recital 15 of the FD). However, the Directive itself does not specify the terms solidarity or fairness, nor how these should be operationalised (Van Eerd et al. 2015). Such an operationalisation is difficult since justice is a social construct (Davy 1997; Martinez-Alier 2012), with different actors attributing different meanings to who should be protected, who should warrant this protection and who should pay for it. Nevertheless, a consideration of these issues is important.

Analytically, a distinction can be made between procedural justice and distributive justice. Procedural justice refers to the fairness of the decision-making process. FRM research is plentiful with debate about adequate and effective participation mechanisms (e.g. Nye et al. 2011; Mees et al. 2016). The focus of this paper, however, is on the second component: distributive justice, referring to the distribution of both the burdens and benefits from FRM. Despite multiple burdens of FRM being distinguished (Penning-Rowsell and Priest 2014), this paper exams two key issues of distributional justice. The burden of flood risk itself, due to differing hydrological circumstances, is inherently unevenly distributed (Johnson et al. 2007; Penning-Rowsell and Pardoe 2012). Furthermore, there is the burden of FRM, i.e. the distribution of responsibilities and the financing of management and recovery strategies (Penning-Rowsell and Pardoe 2014). These burdens are allocated through institutions responsible for managing flood risk. Additionally, flooding is also intrinsically linked to broader social and economic inequities which affect both communities’ and individuals’ resilience, i.e. their ability to prevent damage and recover from flood events. This ability may be diminished for deprived households, single-parent households, elderly or ethnic minorities (Walker and Burningham 2011; Fielding 2012).

Understanding the distribution of current and future burdens is particularly important in the context of worsening flood risks. Probabilities of flooding are projected to increase due to climate change, while consequences are also worsening due to additional socio-economic development in flood-prone areas (De Bruijn and Klijn 2009; Alfieri et al. 2015). These challenges are likely to aggravate issues of justice in FRM.

The Netherlands is an example of a highly vulnerable delta. FRM in the Netherlands has a long tradition with a strongly protectionist approach (Van Heezik 2006). It is characterised by, firstly, the ambition to pre-emptively minimise fluvial and coastal flood probabilities, resulting in one of the most ambitious national safety standards for defence structures in the world (Aerts et al. 2008). Secondly, the financing of this high protection level, annually around one billion Euros, is based on a principle of collective solidarity (Van Rijswick and Havekes 2012). Solidarity implies that the risks and burdens that group members face should be shared by the group as a whole, that is, all inhabitants of the Netherlands (Dawson and Verweij 2012). As such, it implies a (re)allocation of individual flood risk burdens to the collective which is institutionalised into FRM approaches. However, despite the high risks present and the high level of taxes spent on flooding issues, there is little societal or scientific debate about the very principle of this burden sharing. Questions that remain largely undebated include: who carries the burden of FRM? Who is included and excluded from benefiting from the national solidarity approach? Does, and should, the solidarity approach extend to other types of flooding?

Indeed, the OECD (2014: 86) has recognised this issue and raised concerns regarding the fairness of the Dutch solidarity approach, especially if costs for FRM escalate in the future. This paper aims to explore distributional justice in Dutch FRM, based upon the following research questions: (1) how do institutions in the Netherlands allocate the responsibilities and costs for FRM and recovery for different types of flooding? (2) What are underlying conceptions of justice connected to these institutional arrangements? and (3) What are potential challenges with future increasing flood risks?

The second section presents conceptual approaches to analyse institutional arrangements in terms of justice. The methodological approach is explained in section three. Section four describes the distribution of flood risk in the Netherlands, while the following Section five analyses ‘Dutch flood risk management’, i.e. how institutions distribute the burdens for FRM and how the operation of the arrangement creates different distributive justice outcomes. Section six entitled ‘Discourses of FRM in the Netherlands’ studies the explicit conceptions of distributive justice underlying Dutch FRM, and finally, the last section reflects on potential justice challenges for delivering future Dutch FRM.

## Conceptualising approaches to justice in FRM

This paper adopts a discursive institutional perspective (e.g. Hajer 1995; Fischer 2003; Kaufmann et al. 2016). This perspective conceptualises social constructs, such as justice, as discourses. These discourses materialise into institutions that have tangible consequences for all actors by distributing the burdens of flood risk and FRM. This paper defines discourses as ‘specific ensembles of ideas, concepts and categorization […] through which meaning is given to physical and social realities’ (Hajer 1995: 44). Discourses are socially constitutive and embody power, because they influence the way people conceive and value certain problems and suppress alternative conceptions (Foucault 1978). Institutions are defined as ‘the formal or informal procedures, routines, norms and conventions embedded in the organizational structure’ (Hall and Taylor, 1996: 6). Discourses are constitutive of institutions, while simultaneously, discourses are also constituted by the existing institutions (Foucault 1972; Philips and Jorgensen 2002). Even though institutions reflect to some degree particular discourses, e.g. on justice, on flood risk or on governance, a discourse is not par for par translated in an institution. Therefore, similar justice discourses can solidify differently into institutions that, thereby, distribute the burdens for flood risk and FRM differently. Institutions are the intermediary outcomes of an ongoing social process. This process is influenced by the constraining and enabling function of existing institutions (Giddens 1984). It reflects the interaction and domination patterns between actors that may advocate diverging discourses (Hajer 1995).

The paper makes a number of conceptual clarifications and distinctions. The terms justice, fairness and equity are often used interchangeably (Ikeme 2003). For the purpose of clarity, this paper focuses on the concept of distributional justice to align it with the conceptual differentiations of justice in the political philosophy literature (Rawls 1973; Nozick 1974; Mill 2010; Sen 2010). Building on previous research about distributional justice in FRM (Davy 1997; Johnson et al. 2007; Johnson et al. 2008; Thaler and Hartmann 2016), we distinguish four contrasting theoretical perspectives on justice (Table 1).

**Table 1:** Overview of different theoretical understandings of distributive justice

	Elitist	Utilitarian	Rawlsian ‘maximin rule’	Egalitarian
Who is responsible for FRM?	Individual	Major risk: collective → state Minor risk: individual	Collective for the most vulnerable—mainly state	State—collective
Who is benefitting?	Those who can afford it	Protection for the ones that result in highest benefit for society	The most flood-prone, Households with a limited resilience capacity	Everyone has same right to protection
Who pays for FRM?	Beneficiary pays	Collective as long as collective benefits exceed costs	Solidarity based for the vulnerable, Potential individual for those not considered to be the ‘most vulnerable’	Solidarity based

*Elitist/libertarian justice* focuses on the principle of ‘maximum liberty’. It is based on the idea that people are entitled to what they have achieved individually due to their merit or rank and that the government should not intervene (Nozick 1974; Davy 1997). Regarding FRM, this implies that the government does not carry out FRM measures, but that everyone shall carry the burden of flood risk and FRM on their own. This beneficiary pays approach will lead to elitist outcomes because FRM will be dependent on individual capacities, which might be limited to certain elitist groupings. The lack of government intervention also offers room for market actors, such as the establishment of flood insurance, which introduce other ways of spreading burdens between individuals.

*Utilitarian justice* is based on the principle of ‘maximising utility’, that is, redistributing collective resources to achieve the maximum societal benefits (Davy 1997; Mill 2010). Collective vulnerability to flooding is valued above individual vulnerabilities. As a consequence, if flood risk is seen as a collective problem, the state is expected to manage the issue by allocating collective tax income ensuring the maximum utility for the majority. In this conception, solidarity is not a moral obligation, but an economic calculus. If only a minority is exposed to flood risk, a utilitarian perspective would suggest that those ‘at risk’ individually finance FRM as this ensures the greatest utility for the collective majority, i.e. the tax payer.

*Rawlsian ‘maximin rule’* states that: resources should be distributed so that they favour the most vulnerable, i.e. this principle focuses on absolute vulnerabilities (Rawls 1973; Davy 1997). With regard to flooding, two types of vulnerable people can be distinguished: citizens prone to flooding and citizens lacking resilience to flooding, for example financially deprived households. To compensate for the unequal distribution of flood risk, the burden of financing FRM shall be carried by the collective based on solidarity, in contrast to an individual beneficiary pays approach. Taking into account individual responsibility, Kymlicka (2002) distinguished between ‘choice sensitive responsibilities’ (where individuals remain responsible for past choices) and ‘endowment sensitive duties’ (assisting those affected by uncontrollable or unforeseen consequences).^1^ This is important for flood risk managers, who may wish to distinguish between those who have made an active choice to live in known high-flood-risk areas and those who have no choice, where the risk was unknown or has increased due to other changing circumstances.

The *egalitarian principle* builds on the notion of equal opportunity for every citizen in terms of distributional outcomes. It implies a public responsibility to provide a certain level of safety or well-being (Davy 1997; Sen 2010), so it aligns with the idea of the ‘provident’ or ‘providing state’ (Gutmann 1988). In terms of FRM, this could imply that those at risk of flooding should be compensated for any inherent inequalities to obtain the same opportunities as those not at flood risk, and, as such, solidarity-based financing of FRM. In contrast to the ‘Rawlsian maximin rule’ perspective, it is not limited to the most vulnerable, but based on the equal treatment of all citizens, independent of their geographical location, their resilience capacity or other factors, i.e. it focuses on relative vulnerabilities. Institutions shall ensure this equality principle either by providing an equal protection level for everyone or by ensuring that all citizens have equal capacities to ensure their own protection.

## Methodological approach and data analysis

To analyse distributional justice outcomes, the institutional arrangement responsible for Dutch FRM is analysed using the following concepts: rules, actors and resources (Arts and Leroy 2006). These comprehend the formal and informal rules (legislations, procedures and policies) that distribute rights and responsibilities for FRM; the distribution of tasks and competencies between governmental and non-governmental actors; and resources for funding FRM (including taxes and contributions from different types of users). Subsequently, the underlying discourses on justice are analysed, i.e. the conceptions of justice explicitly presented in policy and legal documents, or by governmental authorities.

This paper utilises established social science methodologies, and mixed methods are adopted with a focus on qualitative methods such as policy and discourse analysis (Hajer and Versteeg 2005; Creswell 2013). Data were collected via document analysis (e.g. policy documents, legal texts and secondary literature), which was mainly used to analyse the characteristics of Dutch FRM, in terms of rules, resources and task distribution. This analysis was supported and supplemented by 20 qualitative semi-structured interviews with policymakers and experts, which were mainly used to analyse the discourses, including the understandings and discussions regarding justice. The information from these interviews is mainly paraphrased in the text. Documents and transcripts were systematically coded according to the conceptual framework and the different conceptualisations of justice. Quantitative estimates of risk and FRM financing were sought from a range of sources (national ministerial datasets: e.g. Transport and Water (V&W 2010), its successor Infrastructure and Environment (I&M 2012, 2015); datasets of regional water authorities (UvW 2015); and academic data sources of the research institute COELO). These were supplemented by the aforementioned semi-structured interviews. A distinction was made between low- and high-flood-prone areas in the west and elevated, less-flood-prone areas in the east. However, national spending estimates figures were often lacking or incomplete. Therefore, approximations, expert judgement from interviews or the best case data were used. The lack of data is already a first indication of the absence of justice-based discussions.

## Flood risk in the Netherlands

Three types of flooding are prominent in the Netherlands: coastal, fluvial and, slowly emerging on the political agenda, pluvial flooding. Apart from their causes, they differ in their probabilities, severity and potential impact. Importantly, climate change is projected to affect all three types of flooding by increasing their frequency and/or intensity (De Bruijn and Klijn 2009; Klijn et al. 2012; Verbond van Verzekeraars 2015). The Netherlands is highly prone to fluvial and coastal flooding, due to its location at the delta of four major rivers and due to soil subsidence. A well-recited example of this vulnerability is the storm surge of 1953, which affected large parts of the country and killed more than 1800 people (Van Heezik 2006). Other examples include the high water levels along the rivers Meuse and Rhine in 1993/1995 that triggered the evacuation of over 250,000 people and caused localised flooding (Van Heezik 2006).

Fluvial and coastal flooding has the highest potential impacts, in terms of both damages and fatalities. These flood risks are, however, unevenly distributed geographically (Klijn et al. 2012: 188). Flood probabilities are higher in the delta area, i.e. coastal areas in the west and land bordering the rivers Rhine, Meuse and IJssel. These are also the areas where the economic impact of flooding would be highest. Consequently, these coastal and delta areas are highly protected with around 71% of Dutch primary flood defences situated here (UvW 2015). Altogether, primary flood defences protect 55% of the surface area of the Netherlands and 67% of the population, despite that only 35% of the population are actually prone to flooding (De Moel et al. 2011: 623). The so-called unembanked areas are situated between the source of the flood and a defence structure, i.e. these are areas at risk which are unprotected by primary flood defences. Around 115,000 citizens (<1% of the population) reside in unembanked areas (De Graaf and Van de Veerdonk 2012) which can be found across the whole country, but are mainly in the delta areas of Rotterdam and Dordrecht.

The problem of pluvial flooding started to be recognised in 1998, when large parts of the Netherlands were affected causing damages of €400 million (Jak and Kok 2000). The previously low concern about pluvial flooding means that data about the extent and distribution of pluvial flood risk are limited (interview: expert urban water management). However, annually the insurance industry compensates approximately €90 million for damages from pluvial flooding (Verbond van Verzekeraars 2015).

## Dutch Flood risk management (FRM)

This section presents the institutional arrangements governing pre-emptive FRM and post-event recovery for fluvial, coastal and pluvial flooding and how these practices create different distributive justice outcomes. A key distinction is made between the highly protected areas of the Netherlands and unembanked areas which receive lower, or no, governmental flood protection. A second distinction is the extent to which flood types are treated differently within Dutch FRM, highlighting where burdens are carried within a national solidarity arrangement or by individuals.

### Fluvial and coastal FRM: who benefits and who carries the burdens

#### Pre-emptive FRM: Managing fluvial and coastal flooding using defence structures

In the Netherlands, FRM is the statutory responsibility of the state, which is accountable to ensure the habitability of the country as stipulated in the constitution (Van Rijswick and Havekes 2012). In other words, the states’ role as provider of safety, in egalitarian terms, is highly institutionalised. Specialised national and regional governmental authorities have traditionally constructed, managed and maintained primary flood defence structures. The Rijkswaterstaat, the policy-implementing agency of the Ministry of Infrastructure and Environment, and regional water authorities (established in the Middle Ages and of which there were 23 in 2015) are principally responsible for managing coastal and fluvial flood risk. As a consequence of this long-held high state involvement, citizens’ awareness of flood risk is generally described as low (e.g. OECD, 2014).

Primary flood defences need to fulfil national legal safety standards of a minimum return interval of up to 1 in 10,000 years along the coast and 1 in 1250 years along rivers (Van Rijswick and Havekes 2012). These standards are based on a crude cost–benefit analysis of the economically most important part of the country (dike ring 14) undertaken by the first Delta Committee following the 1953 flood disaster (Van Danzig 1956; Van der Most et al. 2010). The results were translated into protection standards for the primary flood defences and subsequently extrapolated to the rest of the Netherlands (Klijn et al. 2012: 183). The use of cost–benefit analysis implies a *utilitarian* approach to the allocation of protection measures; one might argue that it was not as purely applied as in other countries as it was utilised not to select between options, but to identify protection standards to avoid most damages.

The Delta Programme initiated in 2012 developed new safety standards. These are based upon the provision of a minimal safety level for embanked areas, i.e. a mortality probability of 1 in 100,000 per year. This arrangement implies an *egalitarian* justice principle, or to be more precise, a ‘sufficientarian^2^’ justice principle: the state provides a level of protection so that every citizen enjoys a ‘sufficient’ minimum threshold. Societal cost–benefit analysis compares different management measures (i.e. not only defences are considered but also, for example, provision for evacuation) and their benefit to the collective. These safety standards are used to identify the protection standards for the primary defences. Safety standards can be increased in areas with high potential economic damages, with high population density or where essential infrastructure is located (I&M and EZ 2014: 154). Here, the aim is to ensure highest utility for the majority; thus, in addition to the *egalitarian* characteristics, also *utilitarian* justice principles are present.

In addition to the ca. 3000 km of primary flood defence structures, there are also around 14,000 km of regional flood defences within embanked areas, which provide protection along smaller watercourses (such as the Mark, Lek, Dommel), drainage channels, etc. (Van Rijswick and Havekes 2012). Provinces, in cooperation with regional water authorities, stipulate land use-based safety standards for regional defences: for urban areas, the nationally suggested inundation frequency is 1 in 100 years, and for agricultural areas, it is 1 in 50 years (V&W 1998; Rijksoverheid et al. 2003). Regional standards are lower than for primary defences, since the risk (water depth, velocity, etc.) is generally considered to be lower (STOWA 2004: 10). FRM of this ‘localised’ risk is not institutionalised on the central level, and standards of protection are not equalised across the country, even though steps are undertaken to make the protection more nationally coherent (V&W 1998; UvW and IPO 2004). In this case, a *utilitarian* approach to justice is evident, since the norms are principally based on the economic value of the areas being protected (compare also Boezeman 2015), but due to the flexibility afforded, provinces may decide to provide equal protection standards (e.g. Groningen; see Keessen et al. 2016).

#### Resourcing Dutch FRM: Who pays?

When considering the balance of burdens and benefits of FRM, it is fundamental to analyse resourcing, particularly in the context of substantially increasing costs (Penning-Rowsell and Pardoe 2014). The main investors in fluvial and coastal FRM are the national government and regional water authorities. Data indicate that during the period 2001 to 2014, the costs of FRM have nearly tripled. In 2001, national investments in primary defences were reported to be ca. €400 million; this increased to €800 million in 2009 (V&W 2010: 55) and to over €1 billion in 2014 (I&M 2015: 98). Investment increases might be attributed to improvements resulting from the periodical assessment rounds for primary and regional flood defence structures, which identified that 20 to 30% of assessed structures did not fulfil the legally required standards (Inspectie Verkeer en Waterstaat 2011). Furthermore, in 2012 the Delta Fund was established to finance proactive climate adaptation which corresponds to further spending increases (I&M 2015).

In line with the e*galitarian* understanding of justice, all taxpaying citizens contribute to fluvial and coastal flood protection through primary flood defences. In 2014, the Dutch national income was around 250 billion (Tweede Kamer 2014); of this, less than 1% (~0.4%) was invested in FRM. The main contributions, around 89%, were made by taxpaying households and the rest by businesses (Twynstra Gudde 2015: 30). Taking an average of 2.2 citizens per household (CBS 2015), an average annual household contribution was €100. However, tax contributions vary by income and family situation and, as such, citizens with a higher economic wealth will contribute more. Importantly, individual flood probability is not explicitly considered when calculating national taxpayers’ contributions to FRM, i.e. those at higher risk or defended to a higher standard do not contribute more. It is also necessary to consider household characteristics in flood risk areas. For example, in 2005 the average income in the highly flood-prone (and protected) western provinces was 8% higher than the national average (CBS 2007). This means these citizens will have contributed more to the tax income. Unintentionally, this correlates with their generally higher flood probability and their higher benefit from FRM.

Similar to national investments, the contributions of regional water authorities have also increased in recent years. In 2015, water authorities spent 37% of their budget (i.e. €480 million) on flood defences (Dekking 2015: 10) in comparison with 28% (€268 million) in 2013 (UvW 2014: 44). This almost doubling of FRM spending can be related to the decentralisation of financing. The costs for maintenance and the operation of primary flood defences are no longer subsidised by the national government (interview: regional water authority). Regional water authorities contribute to dike strengthening through the Flood Protection Programme (HWBP, *Hoogwaterbeschermingsprogramma*) which modifies primary flood defences that do not fulfil the legal safety norms (UvW 2014: 44). It foresees a co-financing arrangement consisting of: 50% contribution from national tax revenues; 40% contribution from the collective of *all* regional water authorities (including contributions from authorities without primary defences, i.e. areas that will never directly benefit); and 10% contribution from the specific regional water authority that carries out the FRM task as a so-called efficiency incentive (UvW et al. 2011). The contributions of regional water authorities to primary flood defences are calculated based on the total number of households and the economic value (indicated through the WOZ-value^3^) of an area (Hoeben 2011a: 8). Therefore, regional water authorities with a high economic capacity will contribute more to FRM. Importantly, the probability of flooding or the length of primary defence structures is not directly considered when calculating each authority’s contribution to the programme. Therefore, excluding the 10% efficiency incentive, even though there is a trend for income to be generated more regionally, *egalitarian* principles dominate where costs are spread widely across regions.

Parallel to the national situation, regional water authorities generate their income via taxation. The Water System Tax (*watersysteemheffing*) partially covers the maintenance and management costs of primary flood defences, and nearly the full costs of construction, maintenance and management of regional flood defence structures^4^ (as well as for other water management tasks). The exact allocation of taxes for each task remains unclear. Four categories of taxpaying users are distinguished: households, i.e. citizens residing in an area; property owners, i.e. individuals (citizens and businesses) owning a property; land owners, i.e. mainly farmers or owners of undeveloped land; and nature conservation organisations that manage nature areas. The tax contribution is based on the interest-pay-say principle: the higher the ‘considered’ interest in the management task per category of user, the higher the financial contribution. Population density is seen as an indicator to evaluate the ‘interest’ of households, since in highly urbanised areas, this group benefits most from the work of the water authority. The remaining costs are split across the other three users, whose tariff is based on the economic value of the properties or land, i.e. users with a higher damage potential contribute more (Hoeben 2011b), yet there is no direct link between the contribution and flood probability.

Between regions, the exact contributions differ per category of user. In 2014, property owners (49%) and households (39%) contribute on average proportionally more than land owners (11%) and nature managers (1%) (I&M 2015: 98). The rationale behind this distribution is complex, and several reasons have been proposed. Regional water authorities and farmer interest groups argue it is because urban water management has increased due to ongoing urbanisation and costs are primarily allocated to households as they are seen to benefit most (interview: regional water authority). Other actors, such as citizen interest groups, stress the influence of farmer interest groups in lobbying for greater cross-subsidisation from households to prevent their costs increasing (interview: researcher). Either way, the average annual increase in costs for households from 1998 to 2010 was 7%, for property owners 5.5% from 2000 to 2010, and for land owners, the tariff decreased annually by 0.1% from 1998 to 2010 (Hoeben 2010: 105f). From 2014 to 2015, the average increase in tariffs was: 3.3% for households, 3.4% for property owners and 3.1% for land owners (Allers et al. 2015: 13). Thus, different social categories are treated differently, whereby the differences in contributions cannot explicitly be linked to the flood risk.

Adding to these distributional effects are the differences evident between regional water authorities. These differences do reflect, to some degree, the probability of flooding for collective groups. Households and property owners in the west, with a higher probability of flooding, pay a higher tariff than in the east, with a lower probability. In the east, the average costs to households for flood and water management was around €50/per year in 2015, with property owners (average property value of €211,000) paying an additional ca. €60. In the West, these values are approximately one-third to one-half higher (dataset from COELO). That implies that citizens in areas with a higher flood probability do contribute slightly more to financing FRM, which aligns to some degree with a beneficiary pays understanding. But this is at a collective level, rather than the direct relation of tariffs to individual flood risk probabilities.

#### Post-event recovery

Comparatively, recovery from fluvial and coastal flooding in the Netherlands is considered to be secondary to the high prevention focus. In 1998, the Calamities Compensation Act (CCA, *Wet tegemoetkoming schade bij rampen en zware ongevallen*) was set up to provide post-event compensation. It is solidarity based and aligns to *egalitarian* or *Rawlsian maximin rule* principles. The act is mainly applicable for compensation of damages from freshwater flooding in embanked areas, when an event is declared a disaster, and when no other compensation (e.g. liability) or insurance is available. This implies that citizens affected by coastal (saltwater) flooding, pluvial flooding or citizens living in unembanked areas should not (formally) receive equal governmental support for recovery as citizens affected by fluvial flooding. Therefore, those not covered would be individually responsible for their recovery, implying an *elitist* justice principle (i.e. those outcomes will be better for those who are wealthier or who can afford to access insurance products via the market (*libertarian*)). However, the lack of widely available coastal and fluvial flood insurance (at affordable prices) limits the ability of households to access market mechanisms and reinforces elitist flood recovery outcomes. Furthermore, the government is known to provide compensation in cases where the Act is not applicable, especially for coastal flooding and in unembanked areas (Van Vliet and Aerts, 2014). Accordingly, there is criticism of the government for being ambiguous and creating outcomes based on political will (Botzen and Van den Bergh 2008). This uncertainty creates the potential for those affected by flooding in different circumstances to be treated unequally and leads to different distributive justice outcomes.

#### Unembanked areas: the neglected few

‘Unembanked areas’ are generally excluded from mainstream FRM approaches. Citizens in these areas lack the same opportunities of governmental protection or recovery from fluvial and coastal flooding as citizens residing in embanked areas. However, since the National Delta Programme (2012), more attention is being paid to these areas, for example in Dordrecht, to explore possibilities for flood risk adaptation strategies (I&M and EZ 2014). Even though the probability of flooding in unembanked areas has increased in recent years, the flood risk is generally lower often due to higher elevation (Koks et al. 2015). Citizens in unembanked areas principally have to manage flood risk autonomously and, to do so, need to be aware of flood risks and capable of undertaking measures. In general, regulations do not prevent citizens adapting to flooding as long as measures do not influence discharge capacities; however, incentives for measures are not provided (Van Vliet 2012). Information campaigns to increase citizen risk awareness differ from municipality to municipality leading to high variability of awareness (De Boer et al. 2012).

Even when informed, though, citizens need to be financially able to invest in necessary adaptations. Koks et al. (2015) highlighted that 20% of the inhabitants of unembanked areas in the greater Rotterdam area are considered to be socially vulnerable households, potentially limiting their ability to either adapt to flood risk or to move. Subsidies for private flood-proofing measures to enable deprived households to undertake measures are not systematically provided. This individual responsibility suggests a more *elitist* approach. Furthermore, although around half of regional water authorities offer citizens in unembanked areas a tax reduction (interview: regional water authority), those residing in unembanked areas still contribute to FRM via national and local taxation. It is not true to suggest, however, that those who reside in unembanked areas do not benefit at all from the existence of primary flood defences, but their benefits will be less direct. These residents, for instance, may work in embanked areas or they may benefit from critical infrastructure that is located there. Additionally, they will also benefit from the national economic security afforded by the high flood protection.

### Managing pluvial flooding: a burden of the individual?

#### Pre-emptive pluvial FRM

Pluvial FRM falls largely on the individual. From 2008 onwards, municipalities have a ‘duty of care’ to collect and transport rainwater in public areas. However, importantly, no legal safety standards are nationally prescribed (Gilissen 2013) although technical guidelines foresee, as a rule of thumb, that a sewer has a capacity to flood the street once every two years (Rioned 2006). Municipalities and provinces have discretionary powers to develop and implement management measures for pluvial flooding (Mols and Schut 2012). The management of pluvial flooding can therefore differ between municipalities (ibid).

#### Resourcing pluvial FRM

Pluvial flooding is financed locally, without any regional redistribution (Kunst 2015). The collection and transport of waste water and urban rainwater is financed via the Municipal Sewer Tax (*rioolheffing*). The taxes are mainly generated by households (92%) and to a smaller degree by companies (8%) (Twynstra Gudde 2015) although the specific tariff charged differs between municipalities. Generally, it is a fixed amount for single- or multiple-person households paid by the user or property owner. In some cases, however, the economic value is also considered or it is based on the amount of water consumed. Multiple-person households pay on average €189 (range of €79–€375) for municipal Sewer Taxes (Allers et al. 2015). In recent years, the average tax for households has increased above the level of inflation due to new environmental regulations and the renewal of sewer systems (Allers et al. 2015). The proportion of the tax invested in pluvial FRM differs between municipalities but averages at about one-third (Kunst, 2015: 4). Therefore, across the 393 Dutch municipalities this equated in 2014 to investments of €280 million (excluding VAT) or €20 per inhabitant (Kunst 2015: 4, 15).

Managing this type of flooding is clearly a more individual responsibility and is decided at a municipal level. Consequently, the justice outcomes may be highly variable with those cases a municipality prioritises (perhaps based on where the greatest benefit lies: i.e. a *utilitarian* approach) benefitting greatly and the cases which are not considered residing with the individual, favouring those who are most able to take action (*elitist* justice outcomes).

#### Post-event recovery

Since 1999, insurance for damages from precipitation has been included as part of household (contents and property) insurance. Governmental compensation is formally not foreseen, which implies a more *elitist* and *libertarian* understanding of justice with the individual responsible for purchasing compensation via the private market. Having insurance coverage is often a pre-requisite for having a mortgage, and Spekkers et al. (2013) contend that the market penetration for private insurance is high. However, damage due to rainwater entering the ground floor (e.g. pluvial flooding) requires a supplement. The market penetration rate for this supplement is unknown, as is the average premium cost (interview: Dutch Association of Insurers). In general, premiums are not considered to be risk based due to the bundled nature of the insurance and rainfall-generated damages being only one, often minor, peril covered by the insurance (ibid).

Table 2 summarises the different distributive justice outcomes generated by the institutional arrangements responsible for FRM. It highlights a disparity in how flood risk is managed which varies considerable by flood type and location. Reasons for these differences are explored below, before the potential consequences of these different justice outcomes for the future are discussed in Sect. 7.

**Table 2:** Summary of the distributive justice outcomes of the Dutch institutions responsible for flood risk management

Type of flooding	Coastal and fluvial flooding			Pluvial flooding
	Embanked areas		Unembanked areas	
	Primary flood defences	Secondary flood defences		
Pre-emptive flood risk management	A mix between ***egalitarian (sufficientarian)*** and ***utilitarian*** justice outcomes A minimum basic safety level is a constitutional state responsibility—reinforcing notions of solidarity and *egalitarian* principles. However, differentiated protection standards exist based roughly on the principles of CBA (***utilitarian***). In general, areas with higher coastal risk have higher protection standards for dikes (up to 1:10,000) than those with fluvial flood risk FRM funding—generally from taxation. For national taxes, this shows principally an ***egalitarian*** approach where all pay to reduce the burdens of flood risk. However, for regional taxes (there is a degree of differentiation based on both interests and property values), therefore, this has utilitarian tendencies	Generally an ***utilitarian-***based justice outcome, however, the province can also adopt a more ***egalitarian*** approach (example Groningen) The provinces stipulate protection levels, generally dependent on the economic value behind embankments	***Elitist*** (*Libertarian*) justice outcomes A lack of governmental involvement and assistance necessitates households in unembanked areas to take individual responsibility for their own flood risks and therefore based on individual resources Importantly, no governmental funding is provided to these individuals; however, they do contribute (via taxation) to FRM in the embanked areas	***Mixed*** justice outcomes depending upon the actions of individual municipalities FRM for pluvial flooding is, often, an individual responsibility (*elitist*—i.e. those who can afford to pay for measures) Some municipalities use local taxes to manage pluvial flooding leading to mixed (usually) *utilitarian* or *Rawlsian maximin rule* outcomes depending upon management decisions *Utilitarian*—the municipality takes action where there is most benefit *Rawlsian maximin rule*—the municipality takes action to manage the risk to vulnerable groups (i.e. those least able to help themselves)
Post-event recovery	***Egalitarian*** justice outcomes Fluvial flooding falls under the remit of the CCA, and therefore, the state will provide financial compensation following fluvial flooding (egalitarian). Coastal flooding is formally excluded by the CCA, and therefore, those affected bear the burden for recovery (elitist) as generally insurance is not available or affordable. However, the state may step-in and provide assistance for at least the most vulnerable in society (***egalitarian/Rawlsian maximin rule***)		***Elitist*** justice outcomes Not included within the CCA and so a lack of guaranteed financial compensation The government may offer *ad hoc* assistance (in particular, for vulnerable groups) leading to ***egalitarian*** *or* ***Rawlsian*** ***maximin rule*** outcomes	***Elitist*** (*libertarian*) justice outcomes No government compensation is provided. Citizens are individually responsible for purchasing private market insurance. Therefore, outcomes are dependent on individual resources

## Discourses of FRM in the Netherlands: the undebated issue of justice

A dichotomy is observed in the Dutch FRM approach. The institutional arrangement is characterised by a wide set of regulations that allocate many resources (over €1 billion annually) to the management of (especially fluvial/coastal) flood risk. However, there is hardly any public debate at the national level regarding this distribution. Notions of justice were rarely explicitly discussed during the interviews or in policy documents. This section explores the underlying discourses on justice and assesses whether they correspond with the different distributive justice outcomes.

A foundation of Dutch FRM is that coastal and fluvial flooding is considered an existential threat potentially disruptive to the whole of society. Following the storm surge of 1953, the decision was made that something similar should ‘never’ be allowed to happen again (interviews: national and regional water managers), indicating the very low acceptability of flooding. But these views go even further. The saying ‘the dikes make up the state’ (Elzinga et al. 2006: 171) illustrates that protection from flooding is conceived as a very foundation of the Dutch state. This, in egalitarian terms, provides the state with its legitimacy founded on the notion that it ensures the flood safety of its citizens. Sloterdijk (1998) would label this foundation of Dutch society as the immunisation against the apocalyptic risk of flooding. This makes protection against coastal and fluvial flooding a basic human right and flood defence structures a collective good, essential to maintaining the existence of the Netherlands (interview: Ministry I&M). This protectionist discourse and the low acceptability of fluvial and coastal flood risk indicate why the focus is on pre-emptive FRM at the relative neglect of post-event recovery.

This conception of flood risk is connected to an egalitarian perspective on justice. Interviewees stated that those at risk have the same right to safety as those located in areas less susceptible to fluvial and coastal flooding: those at risk should not be disadvantaged because they live in flood-prone areas as it is not considered to be their fault. This discourse is reinforced in discussions surrounding the new safety norms. It was stated that ‘A human life is worth the same everywhere and the probability of fatality due to flooding must therefore everywhere be fixed at a basic level’ (Deltacommissie 2008: 42). The unequal distribution of flood risk is supposed to be counteracted through FRM. Solidarity is justified by the aim to achieve equal opportunity. Although egalitarian principles dominate FRM, other distributive justice principles have been observed particularly in differences between the west and the east. A number of interviewees stated that citizens in the east are benefiting greatly from industry in the west. As one interviewee put it ‘*it is rather silly that they can live happily on their sand mountain and the money is earned in the west where they risk drowning’* (interview: Ministry I&M). As such, those areas that have higher economic value are provided extra protection illustrating a utilitarian perspective of justice.

This protectionist discourse of solidarity for flooding from the main water courses is characterised by a high consensus. On the one hand, over the decades, the traumatic event of 1953 was retained in the Dutch collective memory. Even younger generations grew up understanding these ‘memories’, e.g. the contrast between new and old buildings in the affected villages, or the dates on the graveyards with many fatalities from 1953 (interview: Ministry I&M). History was constantly reproduced. This transmission of beliefs is contributing to the naturalisation of discourses and the stability of the FRM institutional arrangement (Tolbert and Zucker 1996). The naturalisation of a discourse means that actors start to take particular discourses for granted, while alternative discourses become less accepted. Various governments accept this discourse although it aligns differently with their respective political ideologies: Socialist governments see themselves as protector of the citizens and therefore do not challenge this idea, whereas more liberal parties see flood protection as a necessity for economic growth and as an export product in its own right. Additionally with increasing governmental responsibility and limited flood experience, the awareness of flood risk among Dutch citizens decreased (OECD 2014). Nowadays, Dutch citizens expect the government to manage the risk, which limits their willingness to challenge it. Consequently, political, societal and scientific debate about justice in FRM tends to be limited. All justice discourse became silent (Foucault 1978: 27).

Despite the high consensus and naturalisation of a protectionist approach to FRM, this is not the same for all areas or all flood types; there are clear inequalities in how some individuals and communities at risk of flooding are treated. Egalitarian justice principles are limited to embanked areas. Residents in unembanked areas are considered to ‘*choose on their own to live outside the dike*’ (interviews: national and regional policy makers). In contrast, residents in the embanked areas are not considered to be ‘at fault’, which coincides with a *choice sensitive* discourse, and therefore, they should be individually responsible for the burden of FRM. The notion of choice and fault clarifies the different justice outcomes (embanked/unembanked) generated by the institutions, despite vulnerable households being exposed (Koks et al. 2015).

Likewise, discourses and debates underlying pluvial FRM are different. Local and national governmental authorities view pluvial flooding as a minor problem—a nuisance—that citizens have the capability and responsibility to manage themselves (Rijksoverheid et al. 2003; interviews: Water department of municipalities). Recently, pluvial flooding has received more political attention, mainly at the regional and local level (interviews: Municipalities). A survey of Dutch municipalities revealed that 84% regard pluvial flooding as their most urgent climate-related problem (Wielinga et al. 2015: 29). However, the tolerance of pluvial flooding among municipalities remains higher than for coastal and fluvial flooding (Wielinga et al. 2015: 24; interviews: Municipalities). These differing conceptions of pluvial flooding correspond with the diverse justice outcomes originating from institutional arrangements.

Whereas the discourse on fluvial and coastal flooding became undebated and developed into a ‘silent discourse’ that was naturalised and taken for granted, the justice discourses of pluvial flooding tend to stay largely undebated because pluvial flooding is only slowly starting to emerge as a risk of social relevance. Based on theoretical understandings of Foucault (1978), i.e. that discourses embody power by influencing what receives people’s attention and what stays largely ignored, in this sense, constructing coastal and fluvial flooding as an apocalyptic risk could have contributed to the limited attention paid to other, less deadly or apocalyptic, types of flood risk.

## Conclusion and implications

In general, the existing Dutch system of FRM works effectively and ensures a high level of safety from fluvial and coastal flooding (Klijn et al. 2012: 189). While this system has an abundance of rules, regulations and resources, debates about justice are lacking. The current institutional arrangement has material outcomes that treat particular groups of citizens differently, depending on the type of flooding they are prone to, area they live in (unembanked/embanked) or category of user they belong to (e.g. household, industry, farmer). Accordingly, three individual households with similar flood risk characteristics, in terms of potential probabilities, expected velocities and depths and likely damages, may pay similar financial contributions to FRM, but receive very different outcomes in terms of both pre-emptive FRM and post-event recovery. The access to state-provided FRM differs considerably between citizens prone to coastal/fluvial and pluvial flooding or living in unembanked areas. The latter have few opportunities to seek the enforcement of FRM measures from municipalities, whereas the national government is directly accountable for fluvial/coastal risks. Although the governmental focus on high flood risks is understandable, this may be of little comfort for individuals who have to privately fund their own FRM measures or who suffer the damage and distress caused by flooding and have to finance their own recovery. Especially deprived households, who cannot afford insurance or private FRM measures, are likely to be disadvantaged, thereby increasing existing inequalities, as demonstrated by a study of Penning-Rowsell and Priest (2014: 1006) for the English case.

The debate on justice in FRM is likely to (re)emerge, since the differences in distributional outcomes will become more uneven due to increasing flood risk from climate change, urbanisation and higher economic value (Klijn et al. 2012; KNMI 2015). Maintaining current levels of safety under these conditions means increasing costs for all citizens, either in terms of taxation contribution or private investments in pre-emptive or post-event recovery measures. To some degree, this cost increase for preventing fluvial/coastal flooding is already partly anticipated by the Delta Fund (I&M and EZ 2014). However, due to the high uncertainty, extra investments in the future cannot be excluded, especially considering the ongoing economic development in the flood-prone west (Klijn et al. 2012). Therefore, debates on justice, including where and whom to defend, are likely to emerge, as was the case in England (Johnson et al. 2008), in particular when disparities between expected and affordable protection levels become more obvious.

Potential future debates could include (1) the extension of the national solidarity to all types of flooding and all areas, (2) the withdrawal from national solidarity, or (3) an adaptation of the financial contributions from different groups of users. The extension of the solidarity-based approach might be discussed, if pluvial flooding develops from a local nuisance to a national risk or when citizens in unembanked areas start to be conceived, not as having chosen to live there, but as vulnerable groups (as implied by Koks et al. 2015) that might have been unaware of the risk due to insufficient information (as implied by De Boer et al. 2012). It also might be difficult to sell their land/properties in these areas and move into protected areas, especially for socially deprived households. Conversely, a withdrawal from governmental solidarity could be discussed. The increasing burden on citizens (particularly those only indirectly benefitting from FRM) might lead to a challenge of the appropriateness of maintaining high safety standards through solidarity-based financing. This could be accompanied by a discussion about a more developed private insurance system, considering that the current governmental recovery arrangement is poorly institutionalised (Botzen and Van den Bergh 2008). While this could facilitate economic development in less-flood-prone areas, it might also increase the financial burden for individuals at risk of flooding. Already, the shift from a national to the more regionalised financing structure (e.g. Flood Protection Programme) is projected to cause problems for those regional water authorities (e.g. Zeeland) with many primary flood defences, but which have a lower economic capacity and population density, thereby placing more burdens on flood-prone citizens (Mostert and Doorn 2012). Another discussion might emerge surrounding the distribution of financial contributions of different households and users. These different contributions at national and regional levels are not always based on intentional and explicit justice rationales that consider the differences in capacities or FRM benefits received.

To be prepared for this debate, it is necessary to have the relevant facts and figures concerning the numbers of citizens/households at risk of different types of flooding, their exposures (e.g. water level, velocity), the flood probability, damage potential and (social) vulnerability, as well as market penetration of insurance. Currently, these data are partially lacking. An inclusive public and political debate that allows room to discuss different burdens of FRM and their distribution could strengthen the public support and awareness for flood risk, contributing to more effective and legitimate FRM.

## Footnotes

1 See also the discussion on ‘luck egalitarianism’, which is associated with this distinction (e.g. Knight 2013).

2 In practice not all areas have the *same* standard, but a minimum one, some will be protected to higher degrees.

3 The WOZ-value is the real estate value defined annually by the municipality.

4 The province is also marginally contributing to FRM; however, this is not further elaborated in this paper.
